# Research: Construction and validation of elbow function prediction model after supracondylar humerus fracture in children

**DOI:** 10.1097/MD.0000000000036775

**Published:** 2023-12-29

**Authors:** Qian Wang, Yu Wang, Man He, Haiying Cao, Jingxin Zhao

**Affiliations:** a Department of Orthopedics, Affiliated Hospital of Chengde Medical University, Chengde, Hebei, P. R. China; b Department of Rehabilitation, Affiliated Hospital of Chengde Medical University, Chengde, Hebei, P. R. China.

**Keywords:** nomogram, Stiffness of the elbow joint, supracondylar humerus fracture

## Abstract

This article’s objectives are to develop a model to predict children’s recovery of elbow function following supracondylar fracture, analyze the risk factors affecting those children’s elbow function after surgery, and propose a individualized treatment strategy for elbow function in various children. We retrospectively analyzed clinical data from 410 children with supracondylar humerus fracture. A modeling set and a validation set of kids in the included studies were arbitrarily split into 2 groups on a 7:3 basis. To identify statistically significant risk factors, univariate logistic regression analysis was used. Then, multivariate logistic regression was used with the risk factors, and the best logistic regression model was chosen based on sensitivity and accuracy to create a nomogram; A total of 410 children were included in the study according to the inclusion criteria. Among them, there were 248 males and 162 females, and the fracture type: 147 cases of type IIb and 263 cases of type III. There were no significant changes in the afflicted limb’s lateral difference, surgical method, onset season, and number of K-wires, according to univariate logistic regression analysis. Age (*P* < .001), weight (*P* < .001), height (*P* < .001), preoperative elbow soft tissue injury (OR = 1.724, 95% CI: 1.040–2.859, *P* = .035), sex (OR = 2.220, 95% CI: 1.299–3.794, *P* = .004), fracture classification (Gartland IIb) (OR = 0.252, 95% CI: 0.149–0.426, *P* < .001), no nerve injury before surgery (OR = 0.304, 95% CI: 0.155–0.596, *P* = .001), prying technique (OR = 0.464, 95% CI: 0.234–0.920, *P* = .028), postoperative daily light time > 2 hours (OR = 0.488, 95% CI: 0.249–0.955, *P* = .036) has a significant difference in univariate analysis; Multivariate regression analysis yielded independent risk factors: fracture classification; No nerve injury before surgery; The daily light duration after surgery was > 2 hours; soft tissue injury; Age, postoperative cast fixation time. The establishment of predictive model is of significance for pediatric orthopedic clinicians in the daily diagnosis and treatment of supracondylar humerus fracture.

## 1. Introduction

**Figure 1.**
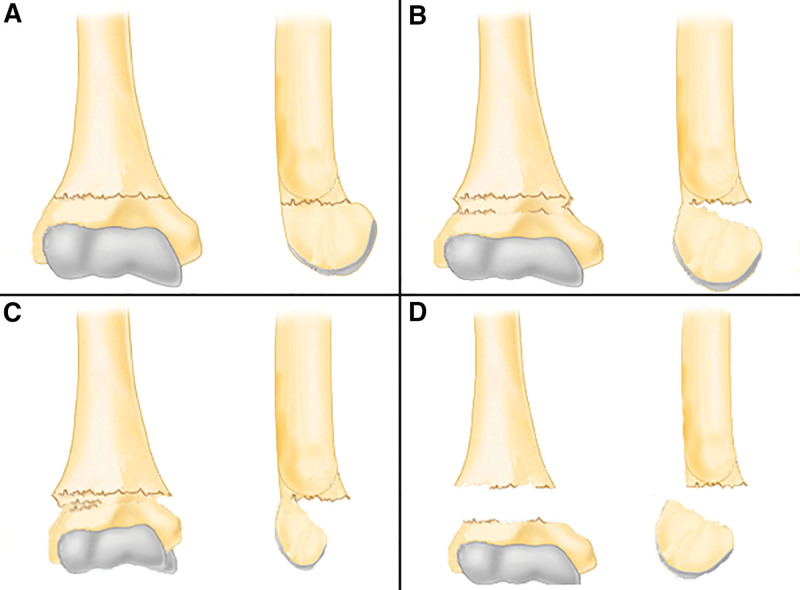
Schematic diagram of Gartland classification of humerus supracondylar fractures. (A) Gartland type I, (B) Gartland type IIa, (C) Gartland type IIb, (D) Gartland type III.

Supracondylar humerus fracture (SHF) is the most common type of elbow fracture in childhood, accounting for 3% of all childhood fractures^[1]^ and 60% of elbow fractures in children.^[2–4]^ It usually occurs in children aged 5 to 10 years,^[5]^ with peak incidence at 5 to7 years old.^[1,5,6]^ According to the posture of the fracture injury, it is divided into extension fracture and flexion fracture. The extension type accounts for 97% of all supracondylar humerus fractures. The extended supracondylar humerus fracture is usually classified into 3 types according to Gartland classification^[7]^: Gartland type I: there is a fracture line but the fracture end is not displaced or the degree of displacement is < 2 mm, and most of them are diagnosed based on the fat pad sign on X-rays; Gartland type II: type IIa: the bone cortex of the anterior condyle of the humerus condyle is broken and the degree of displacement is > 2 mm, the periosteum of the posterior end is intact and the hinge is present, type IIb: based on type IIa, the fracture is rotated; Gartland type III: discontinuity of the bone cortex, complete displacement of the fractured end. (Fig. 1).

Closed reduction and percutaneous pinning (CRPP) is the gold standard of surgical treatment of SHF in children.^[8]^ CRPP has the advantages of short operative time, less trauma, and low risk of surgical site infection. Nevertheless, the presence of risk factors causes fracture healing to take longer than expected, resulting in less optimal postoperative fracture recovery, with elbow dysfunction being the most prominent aspect. The following complications may occur: secondary displacement, premature epiphyseal closure, nonunion of the fracture, nerve damage, valgus deformity of the elbow, and infection of the K-wire tract.^[7]^

At present, relevant studies have been carried out at home and abroad on the risk factors of SHF in children, such as obesity as a risk factor for aggravating supracondylar humerus fracture;^[9–12]^ soft tissue injury location as an independent predictor of vascular nerve injury in supracondylar fractures;^[13]^ the incidence of supracondylar humerus fractures is higher in summer than in other seasons.^[14]^

Although relevant research has suggested pertinent indicators as risk factors for fractures, their clinical usability and effectiveness are far from optimal. Moreover, risk factor analysis of elbow stiffness after SHF is rarely included in the prediction model for visualization. Second, relevant international research mostly focuses on non-Asian populations, and the application of relevant international prediction models to Chinese groups has certain variations due to racial, nutritional, environmental, and genetic variables.

The nomogram is a data graph that uses multivariate regression analysis to show predictors.^[15]^ Regression analysis is used by the nomogram to assign points to predictors. Then, using a function, the total score is converted into the probability that an endpoint event will occur specifically for each individual. Predictive models can successfully identify appropriate “high-risk” groups for the occurrence of endpoint events, aiding clinicians in the early detection of disease and intervention in associated lifestyle habits. They also offer a variety of potential applications in the therapeutic area.

In this study, we build a prediction model that incorporates demographic data, indicators related to hospitalization, and elbow function to predict the likelihood of elbow stiffness following SHF by retrospectively analyzing the risk factors that influence elbow stiffness after supracondylar fracture. Children who were at high-risk for elbow stiffness following supracondylar fracture were identified based on the nomogram indicated by the data, and the model was evaluated for clinical applicability. In a next step, it will serve as a basis for the diagnosis and treatment of certain high-risk children.

## 2. Materials and methods

### 2.1. Study object

Children who underwent surgery with SHF in the Department of Trauma and Orthopedics of the Affiliated Hospital of Chengde Medical University from September 2015 to June 2020 were selected. Identification of inclusion and exclusion criteria, including criteria: Age < 15 years; Clinical diagnosis of supracondylar humerus fracture (Gartland IIb, Gartland III); The clinical data and imaging data of the child are complete; Undergo surgical treatment. Exclusion Criteria: Inclusion in the study cohort was excluded if any of the following were met: Preoperative infection of any part of the body; Multiple fractures or multiple injuries; Malignant tumors, immune dysfunction; There is a local deformity of the elbow joint; Those who have contraindications to surgery; Nonsurgical treatment.

A total of 410 children were included in this study according to the inclusion and exclusion criteria. According to the postoperative review of elbow function, 279 children have divided into the group with normal elbow function and 131 cases the group with elbow stiffness. We have been performed in accordance with the Declaration of Helsinki, got informed consent of legal guardians and have been approved by the ethics committee of Affiliated Hospital of Chengde Medical University.

### 2.2. Research methods

#### 2.2.1. Collection of clinical data.

Age, sex, height, weight, the season of injury, limb disorientation, open fracture status, Gartland classification of fracture, elbow soft tissue injury, presence of nerve injury before surgery, number of K-wires, postoperative plaster fixation time, postoperative daily light time, and elbow joint activity at the most recent follow-up were among the information gathered during hospitalization. Informed consent was obtained from all subjects and/or their legal guardian(s) for the data used.

#### 2.2.2. Surgical methods.

According to the age of the child and the position of the fracture line, the same group of attending physicians decided to perform CRPP. The surgery is performed under general anesthesia. Those who have difficulty in manual reset use the joystick prying technique to assist in resetting.

##### 2.2.2.1. CRPP

**Figure 2.**
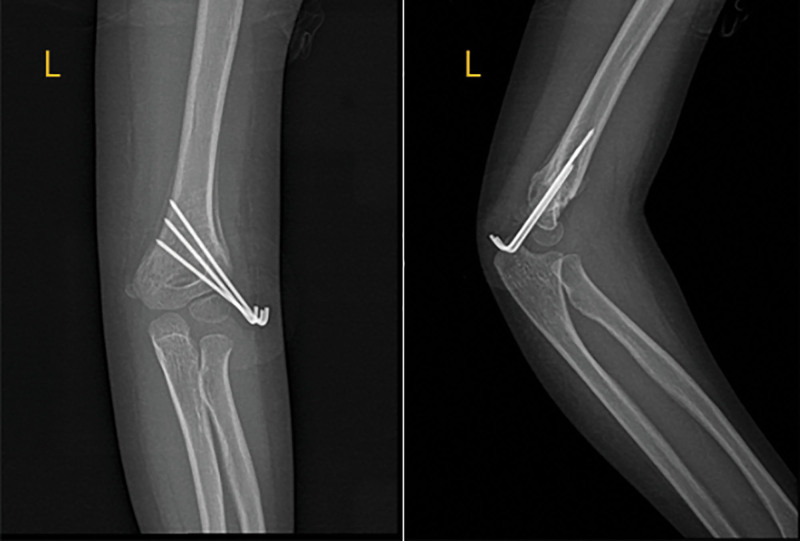
Elbow X-ray after CRPP surgery. CRPP = closed reduction and percutaneous pinning.

Following the child’s effective anesthesia, the patient was placed in the supine position, the operating room’s iodophor was routinely cleaned 3 times, and a sterile cloth was spread out. The surgeon pinches the internal and external condyles of the humerus with 1 hand, correcting the inward or outward displacement of the fractured piece by moving the forearm with the other. This procedure is used when there is mild elbow flexion of 20° to 30°. The assistant pulls the proximal humerus to fight. The assistant helps secure the humeral shaft to correct the rotation of the broken end, slowly bends the elbow joint, and the surgeon applies forward pressure via the thumb to the eagle beak to repair any inward or outward displacement of the fracture block. When the C-arm fluoroscopic humerus orthographic film indicates that the fracture is satisfactorily reduced, the 4 fingers hold down the proximal end of the fracture on the palmar side of the upper arm, fight against the reduction fracture by pronating the palm excessively forward and flexing the elbow for about 120°. They also maintain the forearm immobility. Rotate the humeral shaft to take lateral radiographs of the humerus, and penetrate the K-wires from the outer bottom to the inside and up in the lateral condyle of the humerus, and the broken end of the fracture can be aligned with the line, the direction and length of the K-wires are suitable, and the injured end is stable when the elbow joint is moved, the K-wires is bent and cut short, and the needle tail is left outside the skin. The affected limb is externally fixed with a cast of 90° elbow flexion. (Fig. 2).

#### 2.2.3. Postoperative follow-up.

The child is instructed to go to the pediatric orthopedic clinic of the Affiliated Hospital of Chengde Medical College in the 2nd, 4th, 2^nd^, and 3rd months after the operation, and evaluate and treat possible conditions such as whether wires infection occurs, whether the internal fixation is loose, the plaster cast, the skin sensation of the affected limb, etc., and evaluate the fracture healing by X-ray of the elbow or elbow CT. After the fracture heals, the cast and internal fixation are removed, and at the final review, the recovery of elbow function of the affected limb is assessed and recorded by the Flynn score, Mayo score, and DASH upper extremity function score. Outpatient examinations and evaluations are conducted by 3 senior pediatric orthopedic surgeons. Those with excellent elbow function scores in the last review were classified as normal function, and those with good, fair and poor were classified as the elbow stiffness group.

#### 2.2.4. Model construction and validation.

Study participants were randomly assigned in a 7:3 ratio to a modeling set (281 cases) and a validation set (129 cases). The prediction model for elbow stiffness after condylar fracture of the humerus was created using the data from the modeling set, and then the efficacy and practicality of the model were tested using the data from the validation set. Both data sets were divided into a group with normal function and a group with joint stiffness according to the score of the last postoperative review of the elbow joint for intra- and intergroup validation.

#### 2.2.5. Statistical methods.

The data were statistically analyzed using IBM SPSS 26.0 software (SPSS, Chicago, IL) and Stata 15. The measurement data are tested for normality, and those that conform to the normal distribution are represented by $\bar{x}\pm s$ and those that do not conform to the normal distribution are represented by the median (M). The counting data are expressed in terms of composition ratio (%). Through univariate logistic regression analysis, variables with *P* < .05 were selected, multivariate logistic regression was included, and the model was constructed by the backward stepwise likelihood method. Build multiple sets of models with Stata software. The most valuable logistic regression model was selected as the best prediction model based on the AIC criterion. By visualizing the model data, the predictive model is finally represented as a nomogram. The Receiver operating characteristic curve (ROC) and area under the curve (AUC) were used to evaluate the discrimination ability of the predictive model. The calibration curve is used to determine the calibration ability of the predictive model. The Hosmer-Lemeshow test *P* *˃* .05 indicates that the model has calibration capability.

## 3. Result

### 3.1. General information and variable screening

The children were randomly divided into a modeling set (281 cases) and a validation set (129 cases) using 7:3 numbers. The modeling focused on 188 children with normal elbow function and 93 cases with elbow joint stiffness. The median age was 6 (3,8) years, with the oldest age being 14 years and the youngest age being 1 year; The average height is 115.65 ± 21.37cm; The median body weight was 22 (17.5, 30) kg. In the verification center, 91 children with normal elbow function and 38 cases with elbow stiffness. The median age was 6 (4, 8) years, with the oldest age being 15 years and the oldest being 1 year. There was no significant difference in information between groups. (See Schedule 1 and Schedule 2 for details).

Variable assignments: sex, lateral limb, fracture type (Gartland IIb and Gartland III), preoperative nerve injury, use of joystick technique, and soft tissue injury were defined as dichotomous variables. The postoperative cast fixation time was divided into < 2 weeks; 2 weeks to 4 weeks; > 4 weeks. The number of K-wires was defined as a quasisomy variable. Measurement data include height, weight, and age. The measurement data are grouped by quartile.

Univariate logistic regression analysis: Univariate analysis of the modeling set with postoperative elbow score as the endpoint event showed that there were no significant significances in the lateral differentiation of the affected limb (OR = 1.004, 95% CI: 0.607–1.660, *P* = .989), the season of onset (*P* = .001), and the number of K-wires (*P* = .147). Age (*P* < .001), weight (*P* < .001), height (*P* < .001), preoperative elbow soft tissue injury (OR = 1.724, 95% CI: 1.040–2.859, *P* = .035), sex (OR = 2.220, 95% CI: 1.299–3.794, *P* = .004), fracture classification (Gartland IIb) (OR = 0.252, 95% CI: 0. 149–0.426, *P* < .001), no nerve injury before surgery (OR = 0.304, 95% CI: 0.155–0.596, *P* = .001), using joystick technique (OR = 0.464, 95%CI: 0.234–0.920, *P* = .028), postoperative daily light time > 2 hours (OR = 0.488, 95% CI: 0.249–0.955, *P* = .036) had a statistically significant difference in univariate analysis. (See Table 1).

**Table 1:** Univariate Logistic Regression Analysis of risk factors for children in the training set.

Factor	*P* value	OR	95% CI. For OR	
			Lower	Upper
Sex	.004	2.220	1.299	3.794
The affected limb	.989	1.004	0.607	1.660
Fracture type	˂.001	0.252	0.149	0.426
No nerve injury before surgery	.001	0.304	0.155	0.596
Joystick	.028	0.464	0.234	0.920
The daily light duration after surgery > 2 h	.036	0.488	0.249	0.955
Soft tissue injury	.035	1.724	1.040	2.859
Height	˂.001			
Height (1)	˂.001	13.464	5.934	30.550
Height (2)	˂.001	7.577	3.544	16.198
Height (3)	˂.001	5.100	2.466	10.546
Weight	˂.001			
Weight (1)	˂.001	6.988	3.110	15.704
Weight (2)	˂.001	5.546	2.662	11.555
Weight (3)	.033	2.071	1.059	4.050
Age	˂.001			
Age (1)	˂.001	21.835	9.035	52.772
Age (2)	˂.001	8.318	3.992	17.332
Age (3)	˂.001	20.597	8.147	52.074
Postoperative cast fixation time	.036			
Postoperative cast fixation time (1)	.016	0.656	1.040	1.184
Postoperative cast fixation time (2)	.067	0.742	1.125	1.420
Season of onset	.554			
Season of onset (1)	.182	0.559	0.238	1.314
Season of onset (2)	.344	0.668	0.290	1.541
Season of onset (3)	.586	0.784	0.326	1.883
Number of K-wires	.147			
Number of K-wires (2)	.498	0.657	0.194	2.219
Number of K-wires (3)	.031	2.580	1.092	6.094

### 3.2. Multivariate analysis to build predictive models

The statistically significant risk factors in univariate analysis were as follows: age, weight, height, preoperative elbow soft tissue injury, sex, fracture classification (Gartland IIb type), no nerve injury before surgery, use of joystick technique, postoperative daily light time > 2 hours were included in multivariate logistic regression analysis, forced input method, forward stepwise likelihood method and backward stepwise likelihood method were used respectively to establish a prediction model, and then Stata software was used to compare the advantages and disadvantages between different prediction models. According to the AIC criterion, the best predictive model is selected, that is, the Logistic prediction model obtained by the backward stepwise likelihood method. Fracture classification as independent risk factor was screened (OR = 0.159, 95% CI: 0.076–0.332, *P* < .001); No nerve injury before surgery (OR = 0.365, 95% CI: 0.147–0.910, *P* = .031); The daily light duration after surgery was > 2 hours (OR = 0.463, 95% CI: 0.195–1.102, *P* = .082); soft tissue injury (OR = 1.797, 95% CI: 0.904–3.573, *P* = .094); Age (*P* < .001), postoperative cast fixation time (OR = 3.541, 95% CI: 1.028–12.198, *P* = .032) (Table 2).

**Table 2:** Logistic Regression Analysis Based on the training set.

Factor	*P* value	OR	95% CI.For OR	
			Lower	Upper
Fracture type	˂.001	0.159	0.076	0.332
No nerve injury before surgery	.031	0.365	0.147	0.910
The daily light duration after surgery > 2 h	.082	0.463	0.195	1.102
Soft tissue injury	.094	1.797	0.904	3.573
Age	˂.001			
Age (1)	˂.001	24.956	8.997	69.227
Age (2)	˂.001	10.688	4.529	25.224
Age (3)	˂.001	26.187	9.212	74.441
Postoperative cast fixation time	.032			
Postoperative cast fixation time (1)	.118	0.254	0.045	1.418
Postoperative cast fixation time (2)	.045	3.541	1.028	12.198

### 3.3. Model visualization

**Figure 3.**
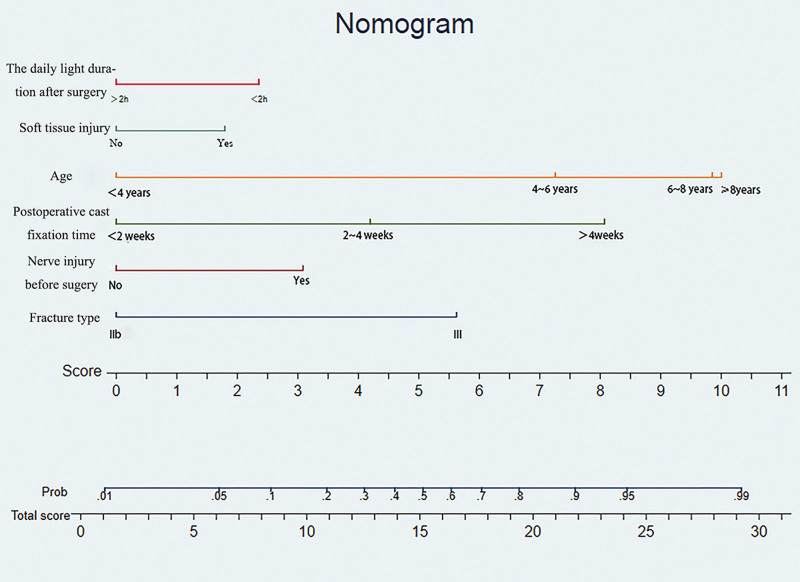
Nomogram for predicting postoperative recovery of elbow function in children with supracondylar humerus fractures.

Based on the data of fracture classification, nerve injury and older children, a prediction model for postoperative elbow stiffness in children with SHF was constructed. Visualize, present, and draw nomogram models. In nomogram, the contribution value of each indicator to the child’s postoperative elbow stiffness is represented by the length of the line segment. How to use nomogram: substitute the indicator information of children with SHF into the chart, obtain the corresponding score at the axis that meets the indicator, summarize all the points, and find the corresponding probability value, which is the probability of the child’s elbow function being damaged after surgery (Fig. 3).

### 3.4. Validate and evaluate the model

#### 3.4.1. The predictive performance of the model.

**Figure 4.**
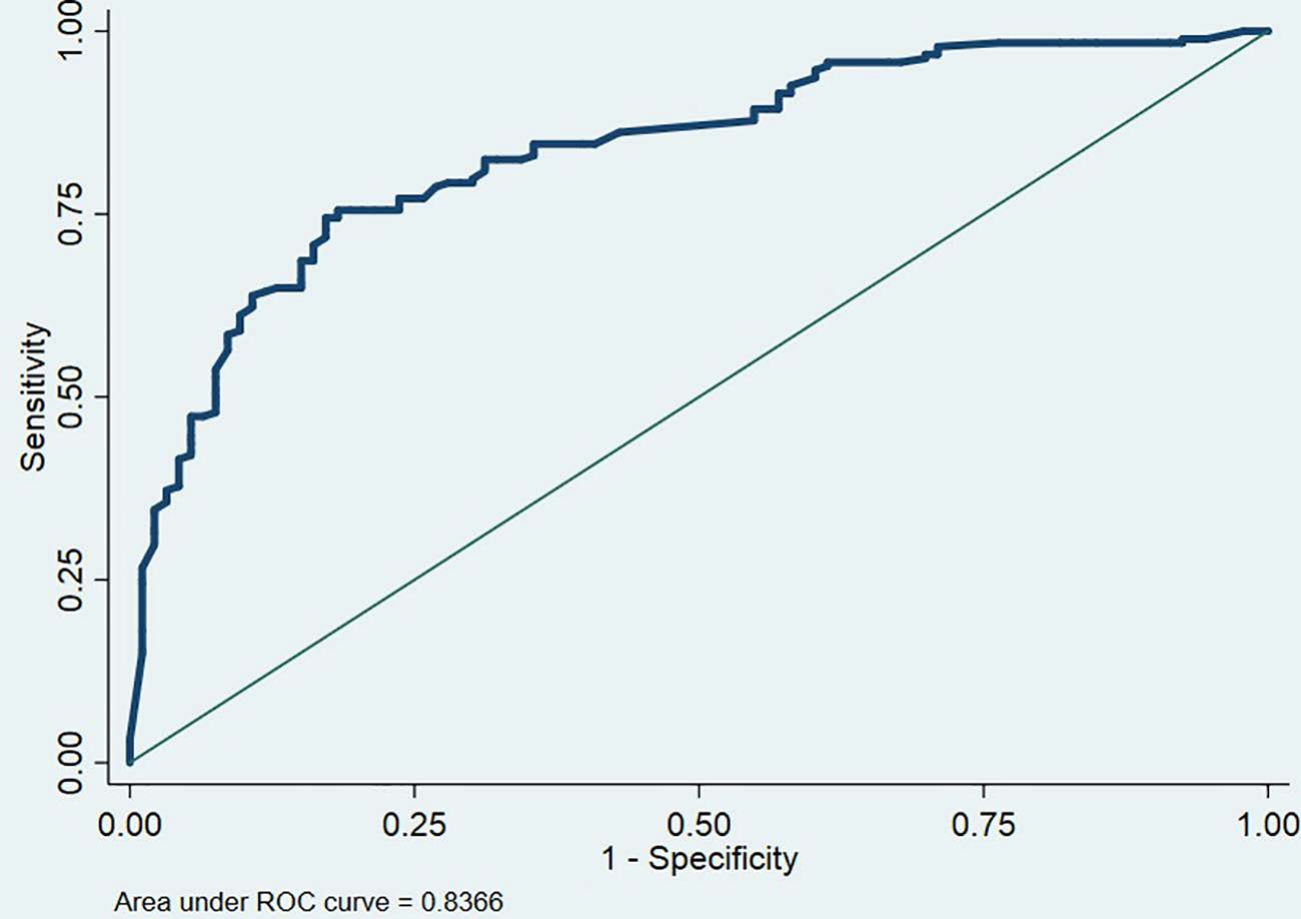
ROC curve of predictive modeling set for postoperative elbow function recovery in children with supracondylar humerus fracture. ROC = receiver operating characteristic curve.

**Figure 5.**
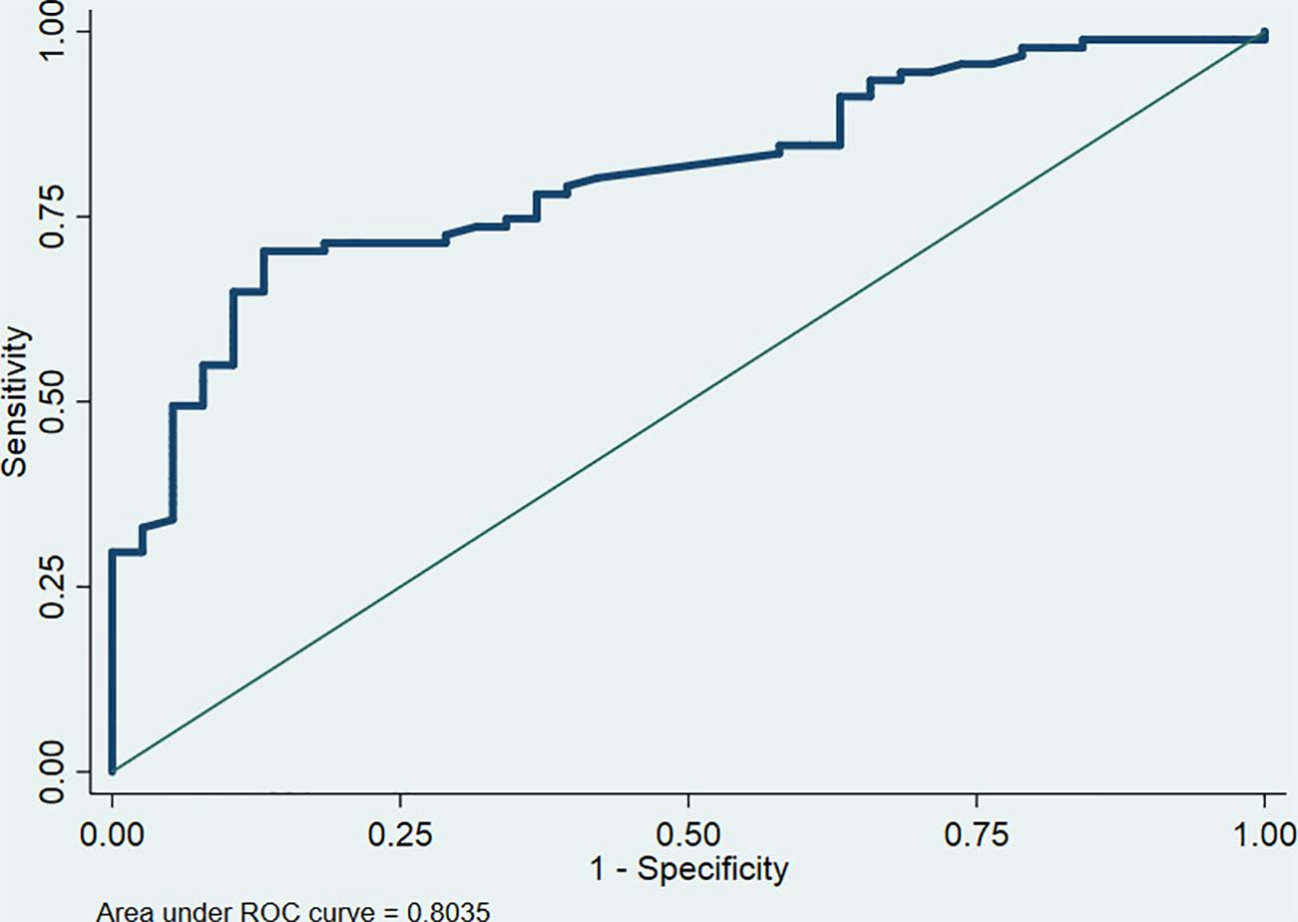
ROC curves of the validation set of predicting risk after surgery in children with supracondylar humerus fracture. ROC = receiver operating characteristic curve.

The ROC curve is one of the main means to judge the performance of predictive models. The closer the AUC is to 1, the better the predictive performance of the model. The results showed that the AUC of the ROC curve that predicted the probability of postoperative elbow function recovery with modeling set data was 0.8366 (95% CI: 0.81–0.90) (Fig. 4). The AUC of the ROC curve of the validation set was 0.8035 (95% CI: 0.79–0.93) (Fig. 5). Both the modeling set and the validation set showed that the predictive performance of this predictive model was better.

#### 3.4.2. Model calibration capability.

**Figure 6.**
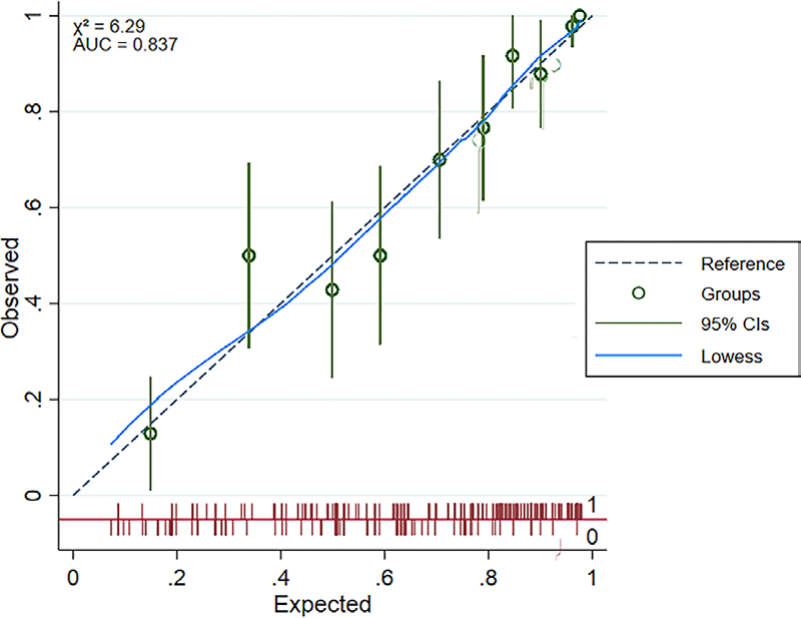
Calibration curve for the predictive model.

Calibration is the difference between the actual observed values and the predicted values of the model. The smaller the variance, the more accurate the model’s predictive power. In the calibration plot, the higher the degree of coincidence between the calibration curve and the ideal curve that crosses the origin and has a slope of 45°, the greater the calibration capability. The results show that the calibration curve fits well with the ideal curve (Fig. 6), and the Hosmer-Lemeshow test shows X^2^ = 6.29 and *P* = .837, indicating that the model has good calibration ability.

#### 3.4.3. Evaluation of clinical practicality.

**Figure 7.**
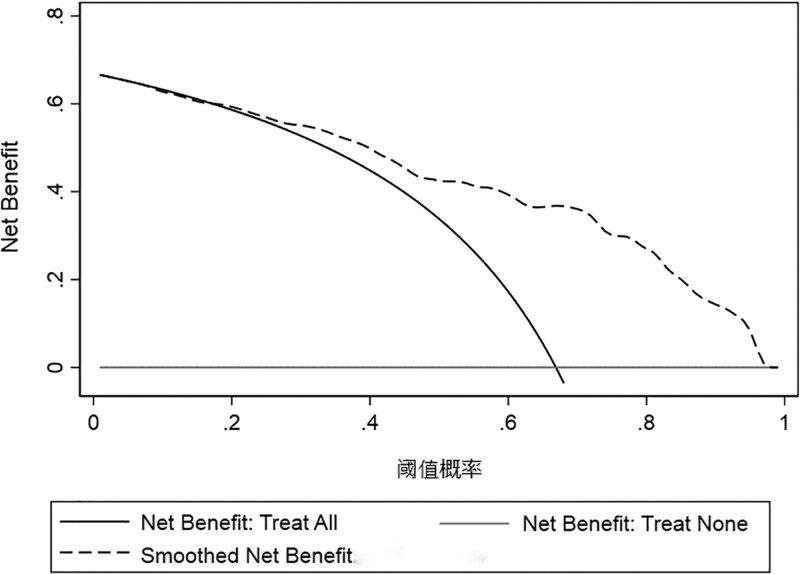
The decision curve analysis of the predictive model.

Decision curve analysis is often used to evaluate the clinical utility of predictive models. The charts mainly include decision curve analysis curves, full benefit curves and non-benefit curves. The area formed by the intersection of the 3 curves is known as the clinical net benefit rate. The results were displayed in the prediction range of 0.2 to 0.98 (Fig. 7), and the prediction of the model was more accurate, and patients can benefit from clinical treatment.

## 4. Discussion

The diagnosis and treatment of SHF in children has long been a hot issue in pediatric orthopedics. One of them is how to reduce the likelihood of elbow stiffness after SHF. To our knowledge, there are few studies that consider elbow stiffness after SHF surgery and predictive models. In order to build a predictive model to determine whether elbow function will recover after surgery in children with supracondylar fractures, we used 13 general baseline information and clinical indicators. The elbow joint score at the last postoperative follow-up was excellently classified into the functional group. According to the results, Gartland type III, nerve injury, daily illumination time < 2 hours, soft tissue injury of elbow, plaster fixation more than 4 weeks, and age can be used as independent risk factors for postoperative elbow function. The treating physician uses this predictive model as a preliminary diagnostic tool to assess elbow stiffness after SHF surgery. The physician then determines the best course of treatment for potential complications.

Due to their high occurrence, SHF is the most frequent elbow fractures in children.^[14,16]^ Because improper treatment can lead to fracture complications and result in significant damage to the child’s health, psyche, and family, appropriate fracture care is essential. Postoperative function of children with supracondylar fractures can be assessed using a variety of methods, including the Flynn score, the Mayo score, and others. However, scoring criteria vary in the clinical context because of differences in definitional criteria and differences in population distribution.

After surgically diagnosing and treating SHF, complications that could result in elbow stiffness include nerve injury, K-wire tract infection, elbow valgus deformity, vascular injury, delayed fracture healing, compartment syndrome, ischemic muscle contracture, and secondary displacement.^[1,6,17–21]^ Iatrogenic injury may also result in nerve damage. Nerve injury can be brought on by fracture displacement, anatomically walking the median nerve anterior to the humeral condyle, medial walking the ulnar nerve, lateral radial nerve, and nerve damage brought on by displacement of the severed end into an angle. After receiving conservative care, the majority of these nerve injuries recover on their own. Thus, nerve exploration by surgery is seldom necessary. In a SHF, Valencia et al^[22]^ reported that the cure rates for nerve injury were 100% for radial nerve injury, 87.5% for median nerve injury, and 25% for ulnar nerve injury, respectively. The median nerve typically took 2.5 months to heal, the ulnar nerve took 5 months, and it took an average of 3 months to fully recover after radial nerve damage. Between 1 to 25% of kids with supracondylar fractures have a K-wire tract infection.^[23]^ The removal of the K-wire and oral antibiotics can treat the majority of superficial K-wire tract infections.^[24]^ Debridement, drainage, and intravenous antibiotic treatment can relieve symptoms in a limited percentage of deep infection or joint involvement instances without leaving behind any serious side effects.^[25]^ Most scholars believe that elbow varus is caused by fracture malunion, not growth arrest. Angular and rotational deformities cause elbow varus. Medial displacement after fracture results in a high Baumann value, which can produce elbow varus deformity, while a lower Baumann value for posterior lateral displacement can lead to elbow valgus deformity.^[26]^ The condition of distal limb perfusion may be a surface indication of extremity vascular patency. The signs can be categorized into 3 groups based on whether a pulse and distal limb blood perfusion are present: Good pulse and good limb perfusion (ultrasonic Doppler detects distal pulse capillary filling < 3 seconds); The pulse is not touched but the hand perfusion is good, that is, the pink pulseless hand (the pulse is not touched, the microvascular filling time is < 3 seconds, and the distal pulse disappears); The pulse is not touched, and the blood flow in the hand is poor (pale, low skin temperature, capillary filling time > 3 seconds). Upper extremity limb ischemia progresses to necrosis and Volkmann ischemic contracture, complications that require immediate surgical exploration in the clinic. Delniotis et al^[27]^ study concluded that for children with pink pulseless hands, after fracture reduction and fixation, the vascular filling state of the affected limb did not deteriorate further, and continued observation was a better choice.

Gartland classification classifies supracondylar fractures of the humerus according to the degree of displacement and stability of the fractured end. Gartland type III is severely displaced and the risk of soft tissue injury and even open fractures is significantly increased.^[28,29]^ At the same time, there is an increased risk of vascular nerve damage and a higher incidence of joint stiffness.^[30,31]^

Nerve damage generally happens as an early side effect of SHF. According to several studies, children have a 10% to 20% chance of developing a supracondylar humerus fracture with nerve damage.^[5]^ The median nerve and the anterior interosseous nerve are the nerves that are most frequently impacted.^[32]^ In this prediction model, 18.7% of children had nerve damage, and both univariate and multivariate logistic regression analysis demonstrated that nerve damage was statistically significant. Avoiding nerve injury as much as you can in the event of humeral supracondylar fractures will aid in a speedy recovery.

Vitamin D plays an important role in growth plate-mediated endochondral osteogenesis and osteoblast-mediated bone synthesis during bone growth and development, and sunlight exposure is the main method for the body to obtain endogenous vitamin D.^[33]^ In our study, daily light < 2 hours was a risk factor for complications of supracondylar fractures of the humerus, and the reasons for this analysis may be related to endogenous vitamin D deficiency and impaired bone health caused by insufficient sun exposure time.

Age > 8 years is defined as older children.^[34]^ Anatomic reduction during surgery is more challenging with older children. The muscles in the child’s upper arm get stronger as they get older, making it easier for them to pull on the fracture’s distal end. The youngster also avoids moving the elbow joint out of dread of pain, which extends fracture healing times and increases the risk of problems including joint stiffness.

As a form of prognostic statistical model, nomogram can not only visually represent the relevant indicators that affect the results of multivariate regression analysis, but also predict the probability of events through simple graphs, which makes prediction easier and more convenient.^[35,36]^ In our study, following many related works,^[37,38]^ a nomogram was constructed and children with SHF were evaluated for risk variables for postoperative elbow stiffness using univariate and multivariate regression analysis. Risk prediction is made possible by nomograms, which depict recognized risk variables. According to the verification results, the model was effective at predicting the likelihood of rehabilitation and discriminating between different scenarios. It has some therapeutic application value as a result.

Some risk factors cannot be controlled, such as the child’s age, the degree of injury, and the presence of concomitant vascular nerve damage, but through the study of these risk factors, we can pay special attention to and treat relevant “high-risk” children, and minimize the adverse effects of risk factors that we can control (such as short light duration or prolonged immobilization of the affected limb). For example, it is recommended that children with fractures appropriately extend the sunshine time, and when the sunlight is insufficient, additional vitamin D should be supplemented to promote the smooth healing of bones. Under the premise of ensuring fracture healing, shorten the immobilization time of the affected limb in children with supracondylar humerus fracture as much as possible and tried not to further increase the damage to soft tissues during fracture reduction, etc. In recent years, we had also made some new attempts in the treatment of supracondylar fractures of the humerus, such as the use of external fixation frames that allow early movement as a surgical method for older children with severely displaced supracondylar fractures of the humerus, to achieve early rehabilitation exercises, which may reduce the occurrence of complications of supracondylar fractures of the humerus.^[39]^

There are several weaknesses in this study. First, only 1 single center was collected in the study population, which may have led to selection bias. Second, the number of cases needs to be increased and more complete cases are collected to improve the current nomogram model. Of course, the risk factors affecting poor recovery of elbow function in children with supracondylar fractures of the humerus are not limited to the ones we have listed, but also include the compliance, personality and mental health of children and parents, parents’ education level and family economic conditions. These factors also play a role in the recovery of elbow function in children with supracondylar humerus fractures. For example, a lively and outgoing child is more active in elbow exercises than an introverted and quiet child, thereby restoring elbow function more quickly. Parents with good education and strong compliance can give better care and guidance in the process of fracture recovery, while families with low education level and poor economic conditions may not be able to adequately take care of children with fractures, thereby increasing the risk of poor prognosis for children with supracondylar fractures of the humerus. These risk factors need to be further explored in future studies.

In summary, in this study, a prediction model of postoperative elbow stiffness in children with SHF was constructed, and the risk factors screened included preoperative nerve injury, fracture type Gartland type III, daily light time < 2 hours, preoperative elbow soft tissue injury, cast fixation time > 4 weeks and older children. The establishment of this prediction model is conducive to the further effective treatment of children with SHF and is conducive to promoting early intervention in postoperative rehabilitation and better promoting the recovery of postoperative elbow function.

## Acknowledgements

We thank all of the patients involved in the study.

## Author contributions

**Conceptualization:** Jingxin Zhao.

**Data curation:** Qian Wang, Man He.

**Formal analysis:** Qian Wang, Yu Wang.

**Funding acquisition:** Yu Wang.

**Investigation:** Qian Wang, Yu Wang, Man He, Haiying Cao, Jingxin Zhao.

**Methodology:** Yu Wang, Man He, Haiying Cao, Jingxin Zhao.

**Resources:** Qian Wang, Man He, Haiying Cao.

**Software:** Qian Wang, Haiying Cao, Jingxin Zhao.

**Supervision:** Yu Wang, Jingxin Zhao.

**Validation:** Qian Wang, Man He, Haiying Cao.

**Visualization:** Qian Wang, Yu Wang.

**Writing – original draft:** Qian Wang, Yu Wang.

**Writing – review & editing:** Yu Wang, Jingxin Zhao.
